# Electromagnetic Fields Due to the Wake of a Moving Slender Body in a Finite-Depth Ocean with Density Stratification

**DOI:** 10.1038/s41598-018-32789-1

**Published:** 2018-10-02

**Authors:** Zhi-Hua Xu, Chang-Ping Du, Ming-Yao Xia

**Affiliations:** School of Electronics Engineering and Computer Science, Peking University, Beijing, 100871 China

## Abstract

Weak electric currents are induced in moving seawater by cutting the geomagnetic fields. These electric currents can produce measurable electromagnetic fields that may be used for some purposes such as monitoring of ocean internal waves. This article is aimed at presenting the procedure to calculate the electromagnetic fields owing to the wake raised by an undersea moving slender body. A pair of Havelock point sources are introduced to model the moving body, which generate the similar wake at places far from the body. The ocean is taken to be of finite-depth with density stratification due to thermocline. Three distinct forms of water-flow wake can be identified, the free-surface Kelvin wake, the internal interfacial wake, and the localized volume wake. The electric currents evoked by the motional wake may produce observable electromagnetic fields, which may be solved using rigorous electromagnetic field theory. At the sea level, the magnitudes of the induced electric field and magnetic field are on the order of a few microvolts per meter and one nano-Tesla, respectively, which are appreciable in terms of nowadays marine electric and magnetic sensors.

## Introduction

The Earth is a huge magnet that produces the geomagnetic field. Just like the motion of a conductor in magnetic field, moving seawater through the geomagnetic field induce motional electric currents, which generate secondary electromagnetic fields. This phenomenon was predicted by Faraday^1^ and quantitatively observed by many researchers^2–4^.

As a kind of motion of seawater, the wakes of ships or submarines can produce electromagnetic fields, and the magnitudes of induced magnetic fields at the sea level are reported on the order of one nano-Tesla (nT)^5–13^. Current commercial optical-pumping atomic magnetometers have a typical precision of a few pico-Tesla (pT) and may be adopted as the sensors. Therefore, it could be tactically significant to detect the electromagnetic fields produced by the wakes of submerged moving vehicles^5,6^.

It is extremely difficult to obtain the exact flow fields or wakes resulting from a real underwater moving body. However, a pair of Havelock point sources may be introduced to model a slender body, which generate the similar wakes at places far from the body^14,15^. The wake generated by a pair of Havelock point sources may be characterized roughly in three forms. The most prominent form is the air-seawater surface wave or the free-surface Kelvin wake, which is a quasi-sinusoidal oscillation and deemed to be a kind of infra gravity waves. It may be regarded as a unique accompanying signature of a moving body, a phenomenon of long distance effect is dominant. The second form is the internal interfacial wave if density stratification due to thermocline exists^16–18^. However, excitation of the internal interfacial wave requires some conditions determined by the Froude numbers, which also determine the behavior of the internal wave as a long-range or short-range traveling wave. The third form is the volume wake localized to the body, which is a direct reflection of pushing-aside and refilling of seawater of the body’s volume.

According to the three forms of wake, the electromagnetic fields induced by the wakes may be described as the three forms, too. However, it seems that only the magnetic anomaly fields induced by the free-surface Kelvin wake has drawn enough attention so far. It seemed that the first simulation results for a ship or a submerged vehicle in an infinite-depth ocean were provided by Madurasinghe *et al*.^7,8^. Extension to stratified case, that is, two-layered but infinite-depth, was presented by Yaakobi^9^ and Chaillout *et al*.^10^. Studies for cases of an infinite-depth ocean (air-seawater), a finite-depth ocean (air-seawater-sediment), a stratified infinite-depth ocean (air-seawater1-seawater2), were also conducted by the present authors^11–13^. In particular, the electric and magnetic fields induced by both the free-surface Kelvin wake and the localized volume wake were discussed^13^. So far, as a more practical model, the case for a finite-depth ocean with density stratification (air-seawater1-seawater2-sediment) has not been considered, which motivates the preset study.

## Simulation Results and Discussion

Refer to Fig. 15 for the model under consideration. Following parameters for simulations are used: *L* = 100 m, *V* = 4*π*×10^4^ m^3^, *z*_0_ = 50 m, *d* = 100 m, *h* = 40 m, *U* = 10 m/s, *ρ*_2_/*ρ*_1_ = 0.974, *σ*_1_ = 5 S/m, *σ*_2_ = 0.1 S/m, *ε*_1_ = 80*ε*_0_ and *ε*_2_ = 8*ε*_0_. The ambient magnetic field is taken to be $|{{\bf{F}}}_{0}|=5\times {10}^{4}$ nT, and ${{\bf{F}}}_{0}/|{{\bf{F}}}_{0}|=(\tfrac{1}{2},0,\tfrac{\sqrt{3}}{2})$ for the geomagnetic coordinate system or the body’s coordinate system if it moves toward the magnetic north pole.

**Figure 1:**
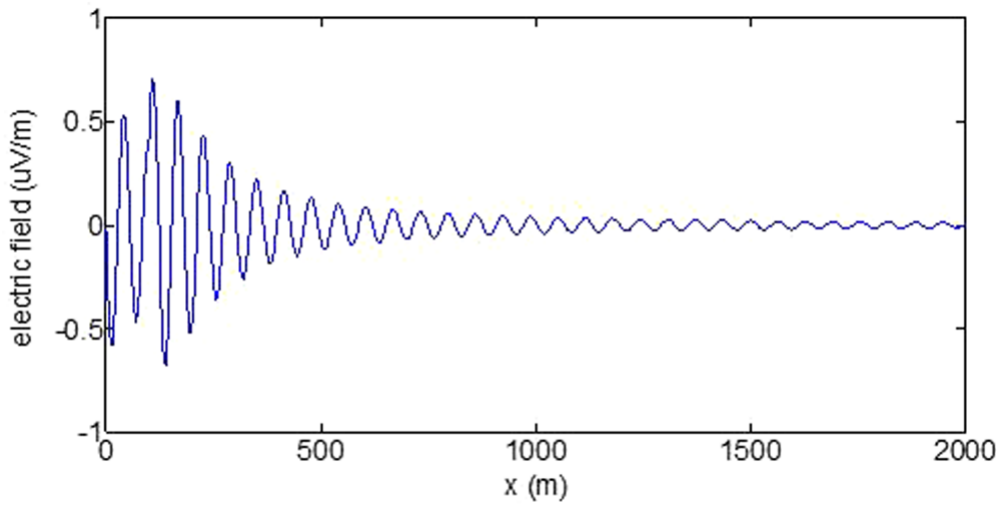
Electric field’s y-component beneath the sea surface, attributed to the free-surface Kelvin wake.

First, the distributions of produced electromagnetic fields at the sea level are calculated. The induced electric fields along the centerline are plotted in Fig. 1 through Fig. 4, corresponding to the free-surface Kelvin wake, internal interfacial wake, localized volume wake, and the full wake (superposition of the three forms of wake), respectively. It is seen that the strength of electric field is on the order of a few microvolts per meter (µV/m), which is a big value in term of current undersea electric field sensor that has a typical precision of several nanovolts per meter (nV/m)^19^. The induced magnetic anomaly fields, which are the projections of the induced vector magnetic fields along the direction of the Earth magnetic field, are shown in Fig. 5 through Fig. 8, corresponding to the three forms of wakes and the full wake, respectively. It is apparent that the magnetic anomaly field is on the order of one nano-Tesla (nT), which is a big value, too, because current commercial optical-pumping atomic magnetometer and marine magnetic sensor possess a typical resolution of several pico-Tesla (pT)^20^.

**Figure 2:**
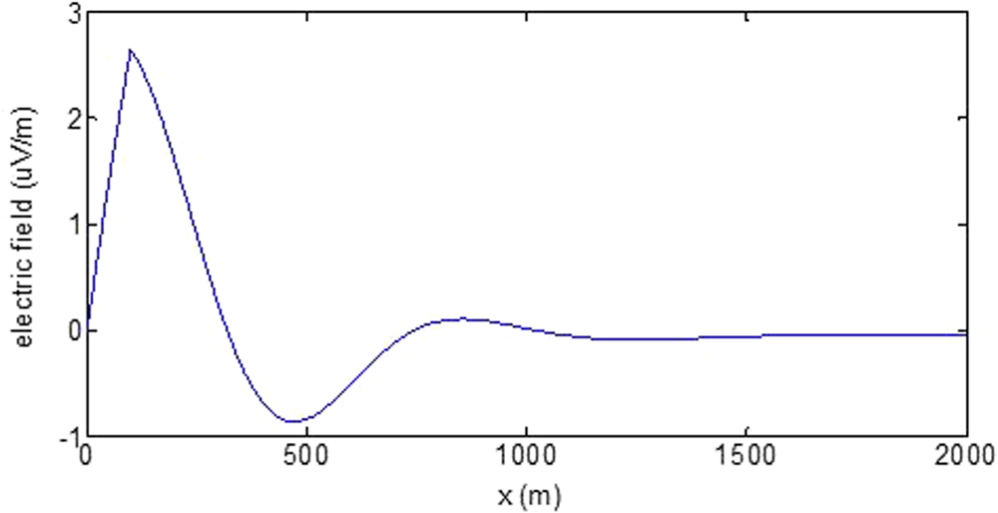
Electric field’s y-component beneath the sea surface, attributed to the internal interfacial wake.

**Figure 3:**
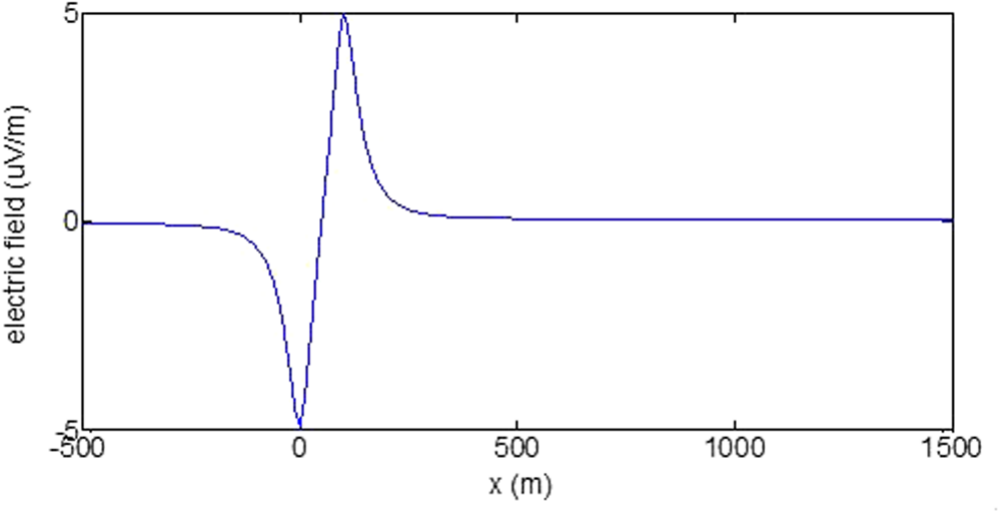
Electric field’s y-component beneath the sea surface, attributed to the volume wake.

**Figure 4:**
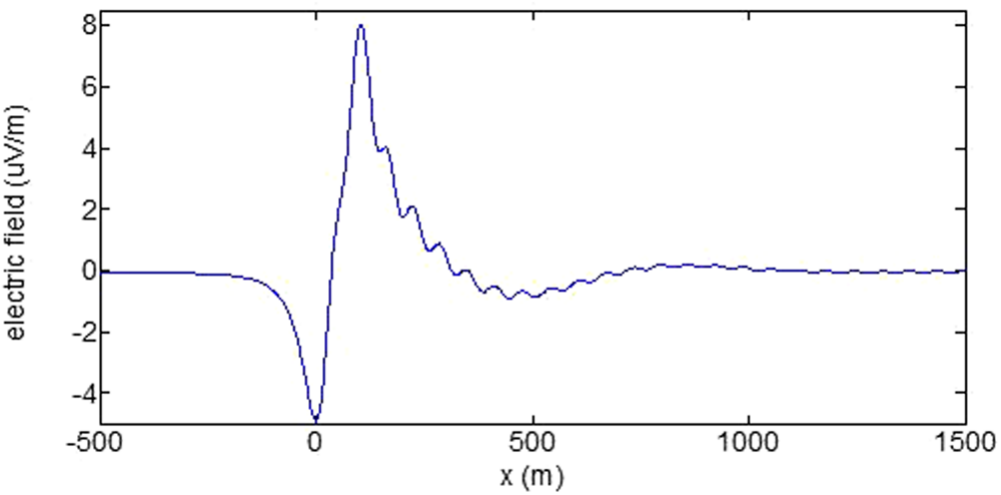
Total electric field’s y-component beneath the sea surface.

**Figure 5:**
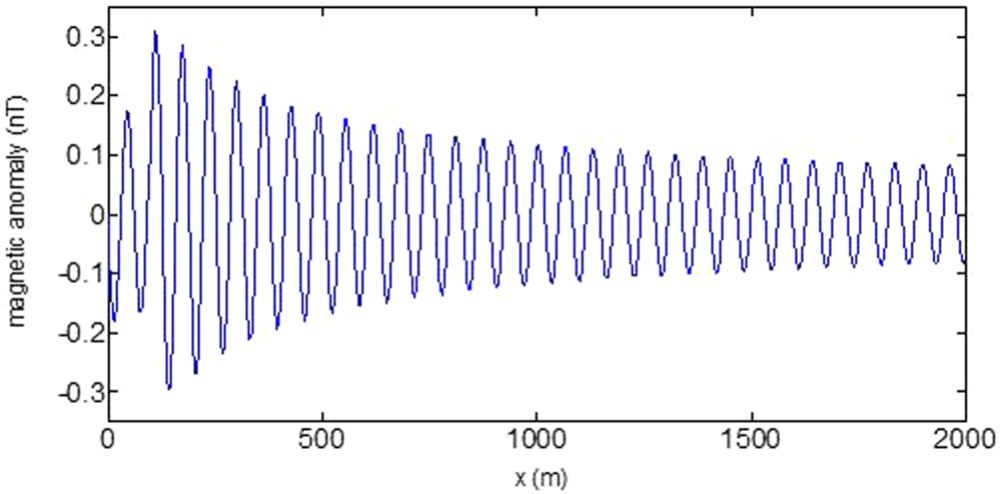
Magnetic anomaly field beneath the sea surface, attributed to the free-surface Kelvin wake.

**Figure 6:**
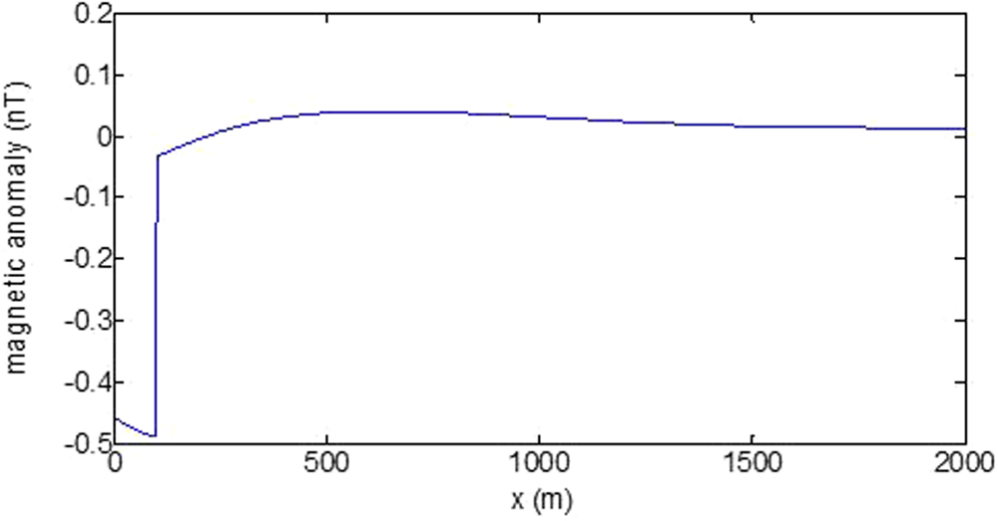
Magnetic anomaly field beneath the sea surface, attributed to the internal interfacial wake.

**Figure 7:**
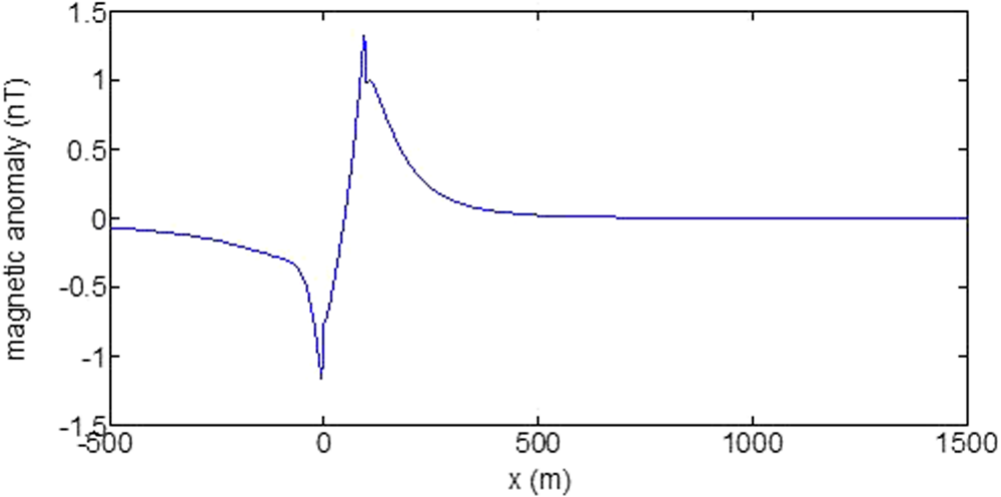
Magnetic anomaly field beneath the sea surface, attributed to the volume wake.

**Figure 8:**
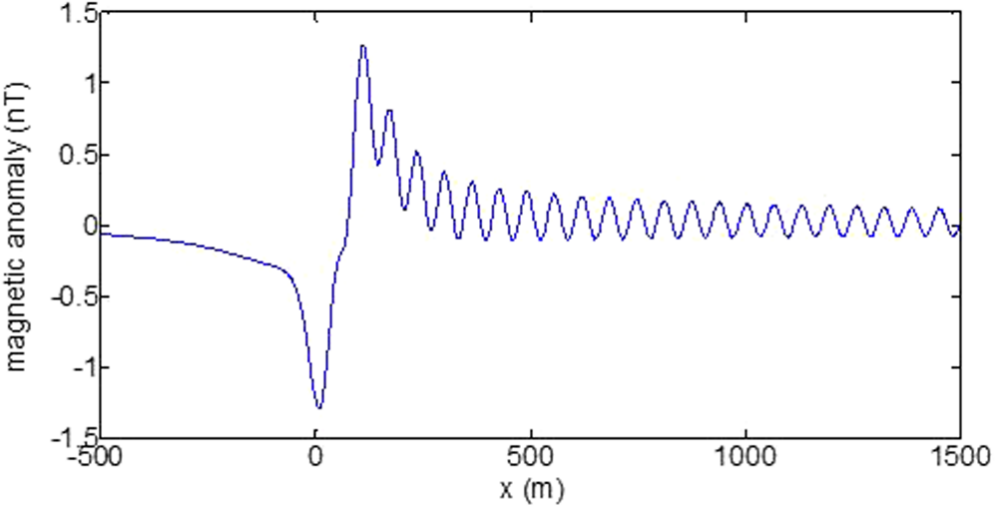
Total magnetic anomaly field beneath the sea surface.

It should be pointed out that we have distinguished the magnetic wakes as three forms according to the three terms in (25). However, in fact, the magnetic wakes are not strictly corresponding to the water-flow wakes that can be fairly characterized as the three forms. For the water-flow, both the free-surface wake and internal-interfacial wake exist only on the side behind of the moving body. However, the magnetic fields induced by the electric currents owing to the two forms of wakes exist not only on the rear side but also on the front side. Thus, one should not over-understand the physical meanings of Figs 1, 2, 3, 5, 6 and 7. What measured are the total fields shown in Figs 4 and 8, where the uneven oscillations on the rear side can be interpreted by the free-surface Kelvin wake.

**Figure 9:**
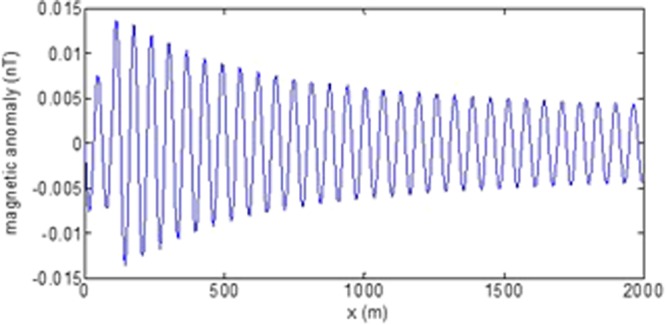
Magnetic anomaly field at 30 m above the sea level, attributed to the free-surface Kelvin wake.

**Figure 10:**
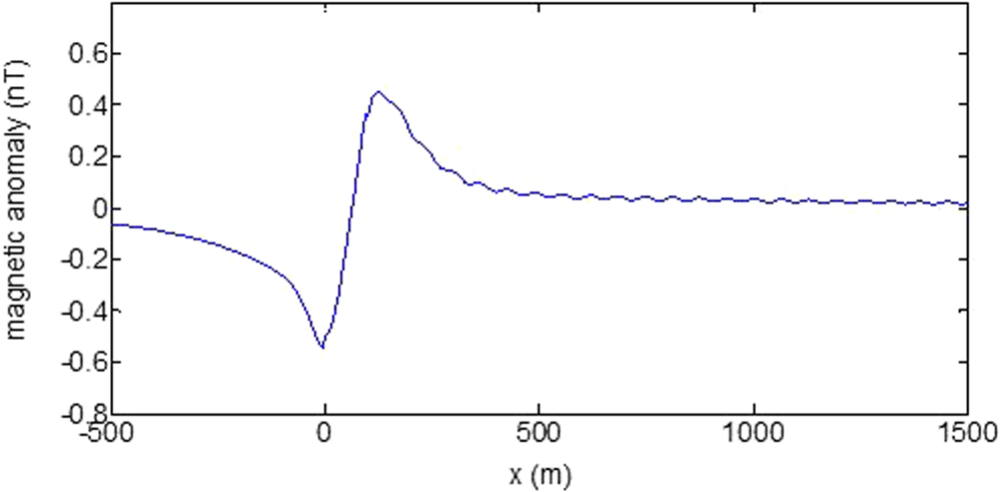
Total magnetic anomaly field at 30 m above the sea level.

**Figure 11:**
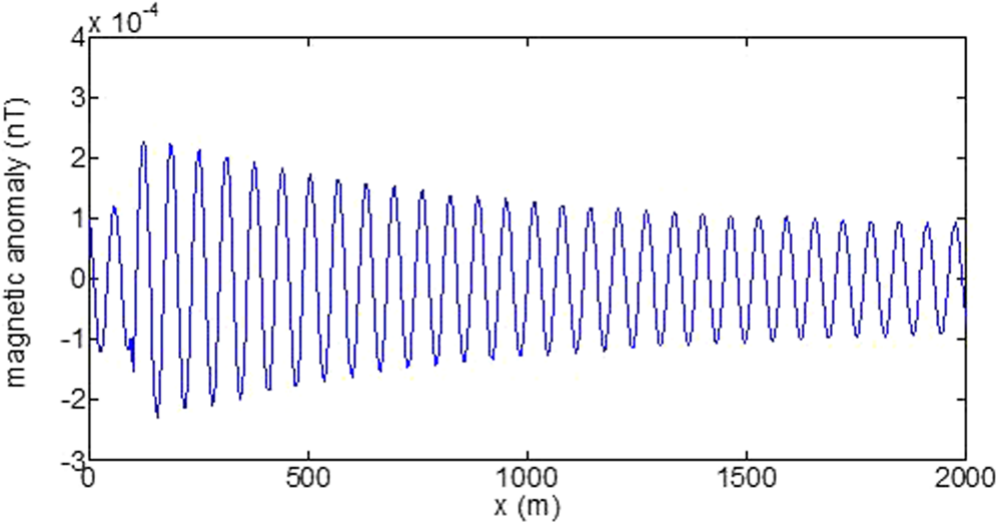
Magnetic anomaly field on the seafloor, attributed to the free-surface Kelvin wake.

**Figure 12:**
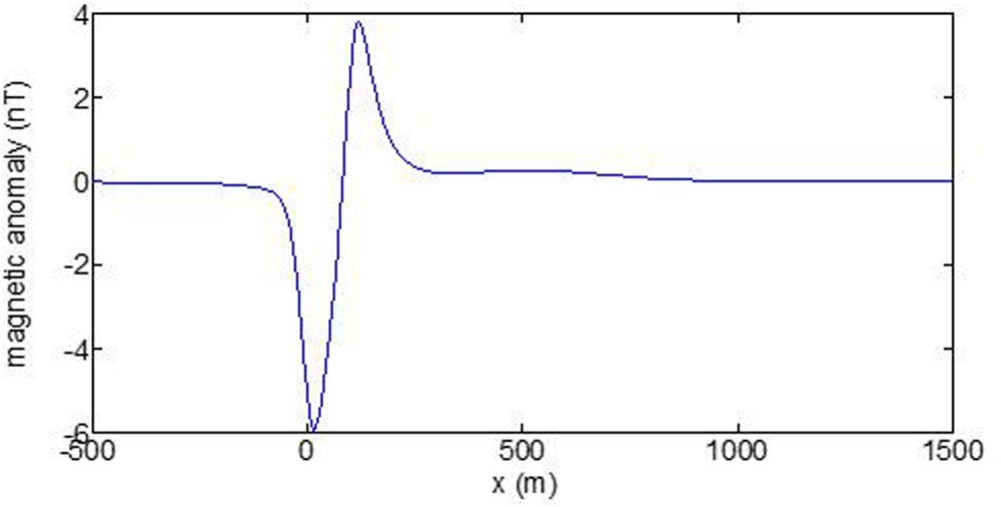
Total magnetic anomaly field on the seafloor.

Next, the induced magnetic anomaly fields at 30 m above the sea level are calculated as shown in Figs 9 and 10, corresponding to the free-surface Kelvin wake and the full wake, respectively. Also, the induced magnetic anomaly fields on the seafloor are calculated and shown in Figs 11 and 12, corresponding to the free-surface Kelvin wake and the full wake, respectively. It is seen that the induced fields attributed to the free-surface Kelvin wake attenuate rapidly in the vertical direction, which is reasonable as the electric currents owing to the water-flow of the surface wake is trapped to the air-seawater surface. Besides, we always plot the induced fields attributed to the free-surface Kelvin wake separately because of its distinct property. The slowly attenuating oscillation may extend over several kilometers and last for a few tens of minutes, which provides a steady signature and may be captured by a narrow-band detector.

**Figure 13:**
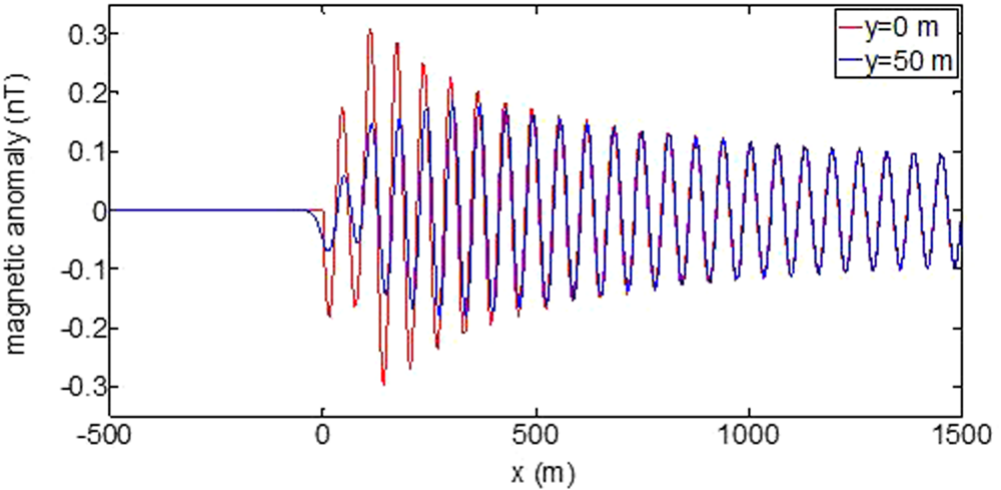
Magnetic anomaly fields beneath the sea surface along two measuring lines, attributed to the free-surface Kelvin wake.

**Figure 14:**
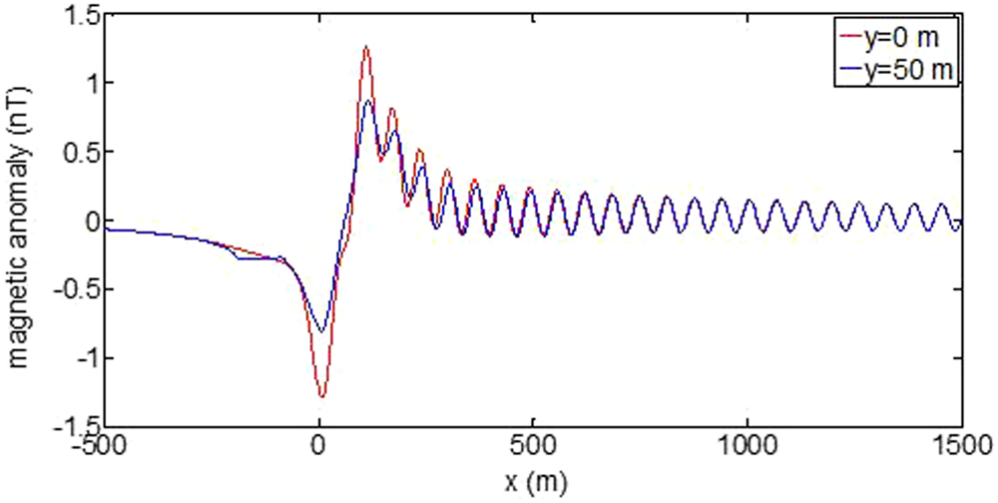
Total magnetic anomaly fields beneath the sea surface along two measuring lines.

Last, the induced total magnetic anomaly fields on the sea surface along two measuring lines, i.e., *y* = 0 and *y* = 50 m, are shown in Figs 13 and 14, respectively, which reflect the transverse attenuating property in the *y* direction. Of course, the induced fields along any a measuring line can be calculated if needed. Again, the induced fields for free-surface Kelvin wake and for the full wake are plotted separately.

## Methods

### Flow Field of Wake

**Figure 15:**
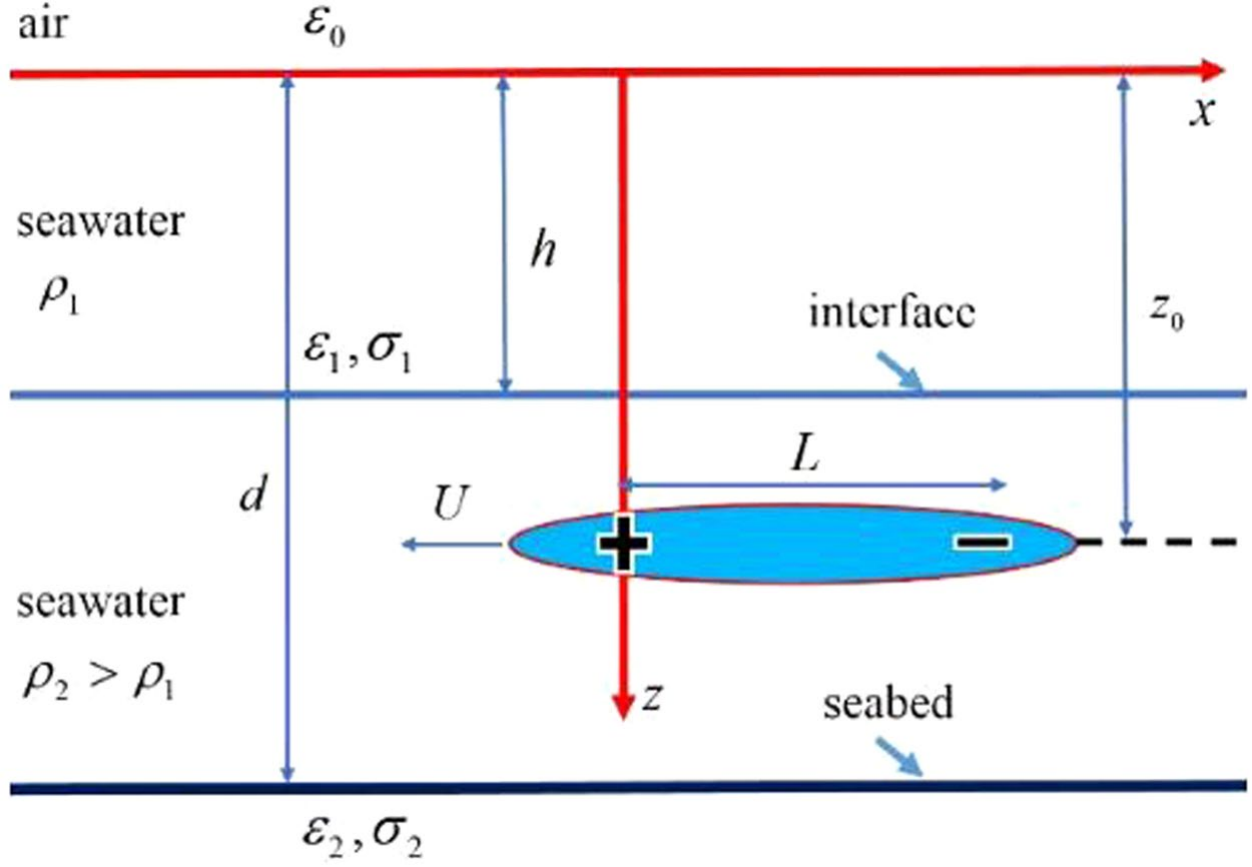
Illustration of a slender body with volume *V* moving at *U* in a finite-depth ocean with density stratification.

A slender body with volume *V* is supposed to move in the lower layer of a two-layered ocean at speed *U* and at depth *z*_0_ as illustrated in Fig. 15. The depth of the upper layer is *h* and the total depth of the sea is *d*. The density ratio of the two-layer seawater is $\gamma ={\rho }_{1}/{\rho }_{2}\le 1$. A source-sink pair separated by *L* of strength ±(*UV*/*L*) can be used to replace the slender body according to hydrodynamics, which generates the similar velocity distribution of water-flow at places far from the body. A velocity potential *φ* can be used to characterize the velocity field by ***v*** = −∇*φ*. Thus, the velocity potential for the pair of point sources can be written as

1 $$\phi (x,y,z,t)=\frac{UV}{L}[G(x+Ut,y,z)-G(x-L+Ut,y,z)]$$

where *G*(*x*, *y*, *z*) represents the Green’s function for an unit-strength point source situated at the origin.

The Green’s functions *G*^(*i*)^(*x*, *y*, *z*), where *i* = 1, 2 refers to the upper layer and lower layer, respectively, should satisfy the following governing equations and boundary conditions^18^

2 $${\nabla }^{2}{G}^{(1)}=0$$

3 $${\nabla }^{2}{G}^{(2)}=-\,\delta (x)\delta (y)\delta (z-{z}_{0})$$

4 $${[{k}_{0}{G}_{z}^{(1)}-{G}_{xx}^{(1)}+\chi {G}_{x}^{(1)}]}_{z=0}=0$$

5 $$\gamma {[{k}_{0}{G}_{z}^{(1)}-{G}_{xx}^{(1)}+\chi {G}_{x}^{(1)}]}_{z=h}={[{k}_{0}{G}_{z}^{(2)}-{G}_{xx}^{(2)}+\chi {G}_{x}^{(2)}]}_{z=h}$$

6 $${[{G}_{z}^{(1)}]}_{z=h}={[{G}_{z}^{(2)}]}_{z=h}$$

7 $${[{G}_{z}^{(2)}]}_{z=d}=0$$

where *k*_0_ = *g*/*U*^2^ and *g* = 9.81 m/s^2^ is the acceleration of gravity; *χ* is the viscosity that will be taken to be zero in this work; *G*_*z*_ = ∂*G*/∂*z*, and so forth. The solutions to (2)–(7) are

8 $${G}^{(i)}(x,y,z)=\frac{1}{{(2\pi )}^{2}}{\rm{Re}}{\int }_{-\pi /2}^{\pi /2}{\int }_{0}^{\infty }{g}^{(i)}(x,y,z;k,\theta )\,dkd\theta ,\,i=1,2$$

where

9 $${g}^{(1)}(x,y,z;k,\theta )=[{C}^{(1)}(k,\theta ){e}^{kz}+{D}^{(1)}(k,\theta ){e}^{-kz}]{e}^{jk{\rm{\Omega }}},\,0\le z < h$$

10 $${g}^{(2)}(x,y,z;k,\theta )=\{{e}^{-k|z-{z}_{0}|}+[{C}^{(2)}(k,\theta ){e}^{kz}+{D}^{(2)}(k,\theta ){e}^{-kz}]\}{e}^{jk{\rm{\Omega }}},\,h\le z < d$$

with

11 $${\rm{\Omega }}={\rm{\Omega }}(x,y,\theta )=x\,\cos \,\theta +y\,\sin \,\theta .$$

The functions *C*^(*i*)^(*k*, *θ*) and *D*^(*i*)^(*k*, *θ*) for *i* = 1, 2 are given in the Appendix. Substituting the Green’s functions into (1), the velocity potential and then the velocity field can be obtained.

## Electromagnetic Fields Due to Wake

First, we consider the electromagnetic fields induced by the fundamental water waves given in (9) and (10). The electric currents in the upper- and lower-layer’s seawater can be fairly approximated by

12 $$\begin{array}{rcl}{{\bf{J}}}_{1}^{(i)}(x,y,z,t) & \approx  & {\sigma }_{1}[-\nabla {g}^{(i)}(x+Ut,y,z;k,\theta )\times {{\bf{F}}}_{0}],i=1,2\\  & = & [{{\boldsymbol{\Lambda }}}^{(i)}(k,\theta ){e}^{kz}+{{\boldsymbol{\Gamma }}}^{(i)}(k,\theta ){e}^{-kz}]{e}^{j(\omega t+k{\rm{\Omega }})}\end{array}$$

where $\omega =kU\,\cos \,\theta$, *σ*_1_ is the conductivity of seawater, and **F**_0_ is the ambient magnetic field. The functions **Λ**^(*i*)^(*k*, *θ*) and **Γ**^(*i*)^(*k*, *θ*) are given in the Appendix. The ${{\bf{J}}}_{1}^{(i)}(x,y,z,t)$ is also a function of *k* and *θ*, but we omit them at the moment and add them back later. The magnetic vector potential produced in the seawater should satisfies

13 $$({\nabla }^{2}-{\mu }_{0}{\sigma }_{1}\frac{\partial }{\partial t}-{\mu }_{0}{\varepsilon }_{1}\frac{{\partial }^{2}}{\partial {t}^{2}}){{\bf{A}}}_{1{\rm{p}}}^{(i)}(x,y,z,t)=-\,{\mu }_{0}{{\bf{J}}}_{1}^{(i)}(x,y,z,t),i=1,2.$$

where the subscript “1p” means the primary potential in the seawater layers. A particular solution to this equation is

14 $${{\bf{A}}}_{{\rm{1p}}}^{(i)}(x,y,z,t)=-\,{\mu }_{0}[{{\boldsymbol{\Lambda }}}^{(i)}(k,\theta )f(z)+{{\boldsymbol{\Gamma }}}^{(i)}(k,\theta )f(\,-\,z)]{e}^{j(\omega t+k{\rm{\Omega }})},i=1,2.$$

with

15 $$f(z)=\frac{{e}^{kz}-{e}^{{\alpha }_{1}z}}{{\beta }_{1}^{2}},\,{[f(z)]}_{U\cos \theta \to 0}=\frac{z}{2k}{e}^{kz}$$

16 $${\beta }_{m}^{2}={\omega }^{2}{\mu }_{0}{\varepsilon }_{m}-j\omega {\mu }_{0}{\sigma }_{m},\,\omega =kU\,\cos \,\theta ,\,{\alpha }_{m}=\sqrt{{k}^{2}-{\beta }_{m}^{2}},\,{\rm{for}}\,m=0,1,2$$

The general/homogeneous solutions in the air, seawater and seabed can be written in the similar forms as:

17 $${{\bf{A}}}_{0}(x,y,z,t)=-\,{\mu }_{0}{{\bf{Q}}}_{0}{e}^{{\alpha }_{0}z}{e}^{j(\omega t+k{\rm{\Omega }})}$$

18 $${{\bf{A}}}_{{\rm{1s}}}(x,y,z,t)=-\,{\mu }_{0}[{{\bf{Q}}}_{1}^{+}{e}^{{\alpha }_{1}z}+{{\bf{Q}}}_{1}^{-}{e}^{-{\alpha }_{1}z}]{e}^{j(\omega t+k{\rm{\Omega }})}$$

19 $${{\bf{A}}}_{2}(x,y,z,t)=-\,{\mu }_{0}{{\bf{Q}}}_{2}{e}^{-{\alpha }_{2}z}{e}^{j(\omega t+k{\rm{\Omega }})}$$

where the subscript “1s” means the secondary potential in the seawater layers. The ${{\bf{Q}}}_{0},\,{{\bf{Q}}}_{1}^{+},\,{{\bf{Q}}}_{1}^{-},\,{\rm{and}}\,{{\bf{Q}}}_{2}$ are constant vectors to be determined by boundary conditions, and given in the Appendix. The total vector potential in the seawater is ${{\bf{A}}}_{1}(x,y,z,t)={{\bf{A}}}_{{\rm{1s}}}(x,y,z,t)+{{\bf{A}}}_{{\rm{1p}}}^{(i)}(x,y,z,t)$ with *i* = 1 and *i* = 2 corresponding to the upper layer and lower layer of the two-layered seawater, respectively.

Finally, the induced electric field and magnetic field are found from the vector potentials as

20 $$[{{\bf{E}}}_{m}({\bf{r}},t),\,{{\bf{B}}}_{m}({\bf{r}},t)]=\frac{1}{{(2\pi )}^{2}}{\rm{Re}}{\int }_{-\pi /2}^{\pi /2}{\int }_{0}^{\infty }[{{\bf{E}}}_{m}({\bf{r}},t;k,\theta ),{{\bf{B}}}_{m}({\bf{r}},t;k,\theta )]dkd\theta ,\,m=0,1,2$$

in which

21 $${{\bf{E}}}_{m}({\bf{r}},t;k,\theta )=\frac{UV}{L}(1-{e}^{-jkL\cos \theta })\{-j\omega {{\bf{A}}}_{m}({\bf{r}},t;k,\theta )+\frac{\nabla [\nabla \cdot {{\bf{A}}}_{m}({\bf{r}},t;k,\theta )]}{{\mu }_{0}({\sigma }_{m}+j\omega {\varepsilon }_{m})}\}$$

22 $${{\bf{B}}}_{m}({\bf{r}},t;k,\theta )=\frac{UV}{L}(1-{e}^{-jkL\cos \theta })[\nabla \times {{\bf{A}}}_{m}({\bf{r}},t;k,\theta )]$$

where the factor $(UV/L)(1-{e}^{-jkL\cos \theta })$ accounts for the pair of Havelock point sources, and the variables (*k*, *θ*) have been added back into the vector potentials.

**Figure 16:**
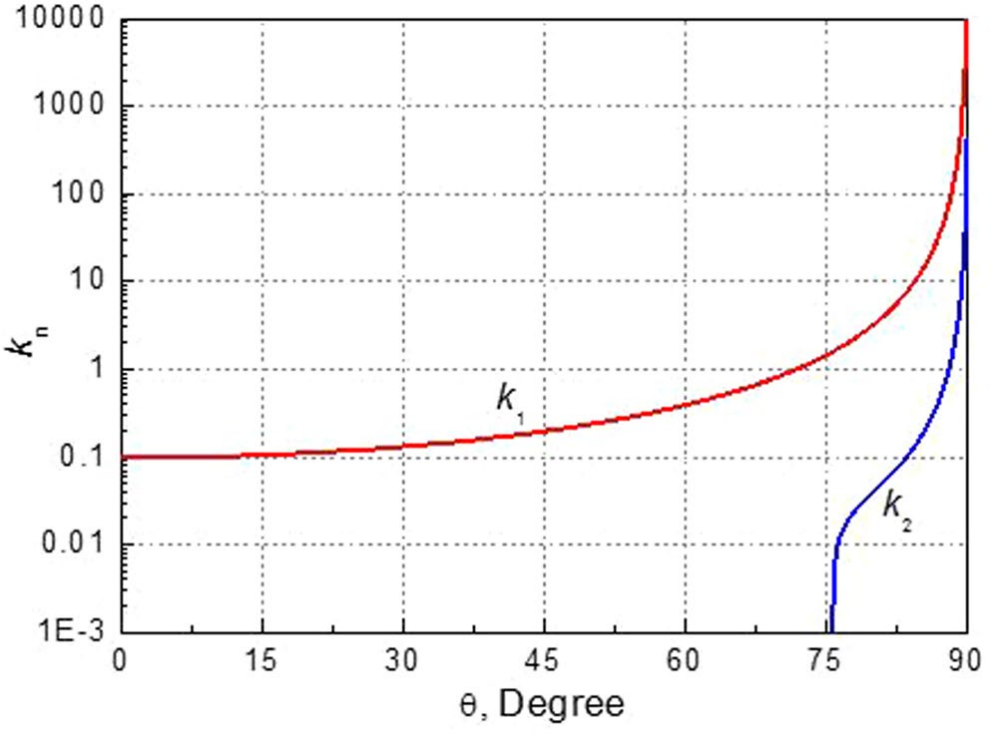
The roots of *H*(*k*, *θ*) = 0 of (31) for given *θ*.

The double integral of (20) will be handled as follows. The integral with respect to *θ* is evaluated numerically at a step size Δ*θ* = *π*/*N* with *N* = 1800. However, the integral with respect to *k* should be treated very carefully. The integrands ${{\bf{E}}}_{m}({\bf{r}},t;k,\theta )$ and ${{\bf{B}}}_{m}({\bf{r}},t;k,\theta )$ are related to **A**_*m*_(***r***, *t*; *k*, *θ*), which would be finally related to *C*^(1)^(*k*, *θ*), *D*^(1)^(*k*, *θ*), *C*^(2)^(*k*, *θ*) and *D*^(2)^(*k*, *θ*) given by (27)–(30). These four coefficients have a common denominator *H*(*k*, *θ*) as given by (31). For a given *θ*, *H*(*k*, *θ*) = 0 would have one root or two roots for *k*, denoted by *k* = *k*_1_(*θ*) and *k* = *k*_2_(*θ*) as shown in Fig. 16. Physically, these two solutions correspond to the air-seawater surface wave mode and the internal interfacial wave mode of the two-layered seawaters, respectively. Whether the two interfacial modes can be created depends on the total depth Froude number *Fr*^2^ = *U*^2^/*gd* and modal Froude number $F{r}_{n}^{2}$ defined by

23 $$F{r}_{n}^{2}=\frac{1}{2}+{(-1)}^{n+1}\sqrt{\frac{1}{4}-\,\frac{(1-\gamma )h(d-h)}{{d}^{2}}},\,n=1,2$$

**Figure 17:**
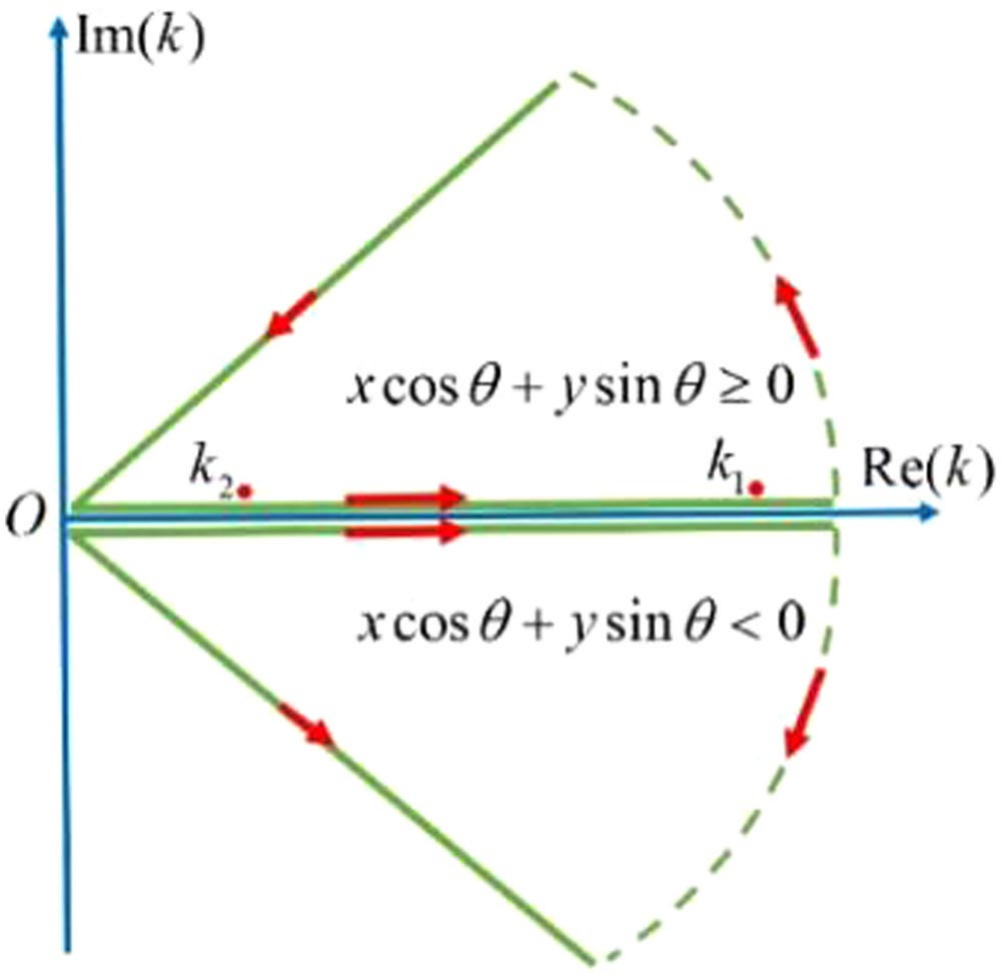
Contour integral schematic diagram.

It can be proved that *k*_*n*_ is present only if $\cos \,\theta  < F{r}_{n}/Fr$. For instance, letting *U* = 10 m/s, *d* = 100 m, *h* = 40 m, and *γ* = 0.974, we have Fr_1_/Fr ≈ 3.122 and Fr_2_/Fr ≈ 0.248. Because $\cos \,\theta  < F{r}_{1}/Fr$ is always satisfied, *k*_1_(*θ*) is present for any *θ*. But *k*_2_(*θ*) is present only for $\cos \,\theta  < F{r}_{2}/Fr$ or 75.63° < |*θ*| < 90°. As a result, the integral with respect to *k* will be performed by using the residue theorem and contour integral as

24 $${\int }_{0}^{\infty }{{\bf{E}}}_{m}({\bf{r}},t;k,\theta )dk=(1-j){\int }_{0}^{\infty }du{[{{\bf{E}}}_{m}({\bf{r}},t;k,\theta )]}_{k=(1-j)u},\,m=0,1,2$$

for $$\tilde{{\rm{\Omega }}}=(x+Ut)\cos \,\theta +y\,\sin \,\theta  < 0$$, and

25 $$\begin{array}{rcl}{\int }_{0}^{\infty }{{\bf{E}}}_{m}({\bf{r}},t;k,\theta )dk & = & (j2\pi )\{{\rm{Res}}{[{{\bf{E}}}_{m}({\bf{r}},t;k,\theta )]}_{k={k}_{1}(\theta )}+{\rm{Res}}{[{{\bf{E}}}_{m}({\bf{r}},t;k,\theta )]}_{k={k}_{2}(\theta )}\}\\  &  & +(1+j){\int }_{0}^{\infty }du{[{{\bf{E}}}_{m}({\bf{r}},t;k,\theta )]}_{k=(1+j)u},\,m=0,1,2\end{array}$$

for $\tilde{{\rm{\Omega }}}\ge 0$. The integral contour is shown in Fig. 17. The residue at *k* = *k*_*n*_ (*n* = 1, 2) is calculated by

26 $${\rm{Res}}{[{{\bf{E}}}_{m}({\bf{r}},t;k,\theta )]}_{k={k}_{n}}={[{{\bf{E}}}_{m}({\rm{r}},t;{k}_{n},\theta )]}_{H(k,\theta )\to H^{\prime} ({k}_{n},\theta )}$$

where *H*(*k*_*n*_,*θ*) → *H*′(*k*_*n*_, *θ*) means replacing *H*(*k*_*n*_, *θ*) by its derivative ∂*H*(*k*_*n*_, *θ*)/∂*k*_*n*_ in the denominators of *C*^(1)^(*k*, *θ*), *D*^(1)^(*k*, *θ*), *C*^(2)^(*k*, *θ*) and *D*^(2)^(*k*, *θ*). The second term in (25) should vanish for $|\theta |\le {\cos }^{-1}(F{r}_{2}/Fr)$. The integral for ${\int }_{0}^{\infty }{{\bf{B}}}_{m}({\bf{r}},t;k,\theta )dk$ is treated in the same way and is not repeated.

## Appendix

The coefficients *C*^(*i*)^(*k*, *θ*) and *D*^(*i*)^(*k*, *θ*) for *i* = 1, 2 in (9, 10) are

27 $${C}^{(1)}(k,\theta )=-\,\frac{2k({k}_{c}-k)}{H(k,\theta )}[{e}^{-k(2d-{z}_{0})}+{e}^{-k{z}_{0}}]$$

28 $${D}^{(1)}(k,\theta )=-\,\frac{2k({k}_{c}+k)}{H(k,\theta )}[{e}^{-k(2d-{z}_{0})}+{e}^{-k{z}_{0}}]$$

29 $${C}^{(2)}(k,\theta )=\frac{4{e}^{-2kd}{\xi }_{1}(k)}{H(k,\theta )}$$

30 $${D}^{(2)}(k,\theta )=\frac{4{e}^{-k(d-h)}{\rm{ch}}[k(d-{z}_{0})]{\xi }_{2}(k)}{H(k,\theta )}$$

with $${k}_{c}={k}_{0}{\sec }^{2}\theta $$, and

31 $$\begin{array}{rcl}H(k,\theta ) & = & ({k}_{c}^{2}-{k}^{2})(\gamma -1)[{e}^{-2k(d-h)}+{e}^{-2kh}]\\  &  & +[{k}^{2}(\gamma +1)-{k}_{c}^{2}(\gamma -1)](1+{e}^{-2kd})-2{k}_{c}k(1-{e}^{-2kd})\end{array}$$

32 $$\begin{array}{rcl}{\xi }_{1}(k) & = & {k}^{2}\{\gamma \,{\rm{sh}}\,(kh){\rm{ch}}\,[k({z}_{0}-h)]+{\rm{ch}}\,(kh)\,{\rm{sh}}\,[k({z}_{0}-h)]\}\\  &  & -{k}_{c}^{2}(\gamma -1){\rm{sh}}\,(kh){\rm{ch}}\,[k({z}_{0}-h)]-k{k}_{c}{\rm{ch}}\,(k{z}_{0})\end{array}$$

33 $${\xi }_{2}(k)={k}^{2}[\gamma {\rm{sh}}(kh)-{\rm{ch}}(kh)]-{k}_{c}^{2}(\gamma -1){\rm{sh}}(kh)-k{k}_{c}{e}^{kh}$$

The **Λ**^(*i*)^(*k*, *θ*) and **Γ**^(*i*)^(*k*, *θ*) for *i* = 1, 2 in (12) are

34 $${{\boldsymbol{\Lambda }}}^{(1)}(k,\theta )=jk{C}^{(1)}(k,\theta ){{\bf{K}}}^{\ast },0\le z < h$$

35 $${{\boldsymbol{\Gamma }}}^{(1)}(k,\theta )=jk{D}^{(1)}(k,\theta ){\bf{K}},0\le z < h$$

36 $${{\boldsymbol{\Lambda }}}^{(2)}(k,\theta )=\{\begin{array}{ll}jk[{C}^{(2)}(k,\theta )+{e}^{-k{z}_{0}}]{{\bf{K}}}^{\ast }, & h\le z < {z}_{0}\\ jk{C}^{(2)}(k,\theta ){{\bf{K}}}^{\ast }, & {z}_{0}\le z\le d\end{array}$$

37 $${{\boldsymbol{\Gamma }}}^{(2)}(k,\theta )=\{\begin{array}{ll}jk{D}^{(2)}(k,\theta ){\bf{K}}, & h\le z < {z}_{0}\\ jk[{D}^{(2)}(k,\theta )+{e}^{k{z}_{0}}]{\bf{K}}, & {z}_{0}\le z\le d\end{array}$$

with

38 $${\boldsymbol{K}}={\sigma }_{1}{{\bf{F}}}_{0}\times (\hat{\eta }+j\hat{z}),\,{{\boldsymbol{K}}}^{\ast }={\sigma }_{1}{{\boldsymbol{F}}}_{0}\times (\hat{\eta }-j\hat{z}),\,{\rm{and}}\,\hat{\eta }=\hat{x}\,\cos \,\theta +\hat{y}\,\sin \,\theta $$

The ${{\bf{Q}}}_{{\rm{0}}},{{\bf{Q}}}_{1}^{+},{{\bf{Q}}}_{1}^{-},{\rm{and}}\,{{\bf{Q}}}_{2}$ in (17)–(19) are determined by the boundary conditions of tangential continuities of both the electric field and magnetic field over the air-seawater and seawater-seabed interfaces, which amount to enforcing the continuity of the four quantities: **A**_*t*_, ∂**A**_*t*_/∂*z*, *A*_*z*_ and *ψ* = ∇⋅**A**/(*σ* + *jωε*), where **A**_*t*_ and *A*_*z*_ are respectively the tangential components and z-component of **A**, and *ψ* is known as the electric scalar potential. As a result, **Q**_0_ is written as ${{\bf{Q}}}_{0}={{\bf{Q}}}_{0t}+{Q}_{0z}\hat{z}$, and so are ${{\bf{Q}}}_{1}^{+},{{\bf{Q}}}_{1}^{-},{\rm{and}}\,{{\bf{Q}}}_{2}$. The eight equations to determine the eight unknowns ${{\bf{Q}}}_{0t},\,{{\bf{Q}}}_{1t}^{+},\,{{\bf{Q}}}_{1t}^{-},\,{{\bf{Q}}}_{2t},\,{Q}_{0z},\,{Q}_{1z}^{+},\,{Q}_{1z}^{+},\,{\rm{and}}\,{Q}_{2z}$ are as follows:

39 $${{\bf{Q}}}_{0t}={{\bf{Q}}}_{1t}^{+}+{{\bf{Q}}}_{1t}^{-}$$

40 $${\alpha }_{0}{{\bf{Q}}}_{0t}={\alpha }_{1}({{\bf{Q}}}_{1t}^{+}-{{\bf{Q}}}_{1t}^{-})+{{\boldsymbol{\Lambda }}}_{t}^{(1)}{Y}_{1}-{{\boldsymbol{\Gamma }}}_{t}^{(1)}{Y}_{1}$$

41 $${{\bf{Q}}}_{2t}{e}^{-{\alpha }_{2}d}={{\bf{Q}}}_{1t}^{+}{e}^{{\alpha }_{1}d}+{{\bf{Q}}}_{1t}^{-}{e}^{-{\alpha }_{1}d}+{{\boldsymbol{\Lambda }}}_{t}^{(2)}f(d)+{{\boldsymbol{\Gamma }}}_{t}^{(2)}f(\,-\,d)$$

42 $$-{\alpha }_{2}{{\bf{Q}}}_{2t}{e}^{-{\alpha }_{2}d}={\alpha }_{1}({{\bf{Q}}}_{1t}^{+}{e}^{{\alpha }_{1}d}-{{\bf{Q}}}_{1t}^{-}{e}^{-{\alpha }_{1}d})+{{\boldsymbol{\Lambda }}}_{t}^{(2)}{Y}_{2}+{{\boldsymbol{\Gamma }}}_{t}^{(2)}{Y}_{3}$$

43 $${Q}_{0z}={Q}_{1z}^{+}+{Q}_{1z}^{-}$$

44 $${\varepsilon }_{1r}^{\ast }(jk\hat{\eta }\cdot {{\bf{Q}}}_{0t}+{\alpha }_{0}{Q}_{0z})=[jk\hat{\eta }\cdot ({{\bf{Q}}}_{1t}^{+}+{{\bf{Q}}}_{1t}^{-})+{\alpha }_{1}({Q}_{1z}^{+}-{Q}_{1z}^{-})]+{{\rm{\Lambda }}}_{z}^{(1)}{Y}_{1}-{{\rm{\Gamma }}}_{z}^{(1)}{Y}_{1}$$

45 $${Q}_{2z}{e}^{-{\alpha }_{2}d}={Q}_{1z}^{+}{e}^{{\alpha }_{1}d}+{Q}_{1z}^{-}{e}^{-{\alpha }_{1}d}+{{\rm{\Lambda }}}_{z}^{(2)}f(d)+{{\rm{\Gamma }}}_{z}^{(2)}f(\,-\,d)$$

46 $${\varepsilon }_{1r}^{\ast }(jk\hat{\eta }\cdot {{\bf{Q}}}_{2t}-{\alpha }_{2}{Q}_{2z}){e}^{-{\alpha }_{2}d}={\varepsilon }_{2r}^{\ast }\{\begin{array}{c}jk\hat{\eta }\cdot ({{\bf{Q}}}_{1t}^{+}{e}^{{\alpha }_{1}d}+{{\bf{Q}}}_{1t}^{-}{e}^{-{\alpha }_{1}d})+{\alpha }_{1}({Q}_{1z}^{+}{e}^{{\alpha }_{1}d}-{Q}_{1z}^{-}{e}^{-{\alpha }_{1}d})\\ +jk\hat{\eta }\cdot [{{\boldsymbol{\Lambda }}}_{t}^{(2)}f(d)+{{\boldsymbol{\Gamma }}}_{t}^{(2)}f(-d)]+{{\rm{\Lambda }}}_{z}^{(2)}{Y}_{2}+{{\rm{\Gamma }}}_{z}^{(2)}{Y}_{3}\end{array}\}$$

In the above, ${\varepsilon }_{mr}^{\ast }=({\varepsilon }_{m}-j{\sigma }_{m}/\omega )/{\varepsilon }_{0}$ for *m* = 0, 1, 2, ${\Lambda }_{z}^{(2)}=\hat{z}\cdot {{\boldsymbol{\Lambda }}}^{(2)}$, ${{\boldsymbol{\Lambda }}}_{t}^{(2)}={{\boldsymbol{\Lambda }}}^{(2)}-{{\boldsymbol{\Lambda }}}_{z}^{(2)}\hat{z}$, and

47 $${Y}_{1}=\frac{k-{\alpha }_{1}}{{\beta }_{1}^{2}},\,{Y}_{2}=\frac{k{e}^{kd}-{\alpha }_{1}{e}^{{\alpha }_{1}d}}{{\beta }_{1}^{2}},\,{Y}_{3}=-\,\frac{k{e}^{-kd}-{\alpha }_{1}{e}^{-{\alpha }_{1}d}}{{\beta }_{1}^{2}}$$

Let

48 $${\alpha }_{1}{{\bf{T}}}_{1}={{\boldsymbol{\Lambda }}}_{t}^{(1)}{Y}_{1}-{{\boldsymbol{\Gamma }}}_{t}^{(1)}{Y}_{1},\,{{\bf{T}}}_{2}={{\boldsymbol{\Lambda }}}_{t}^{(2)}f(d)+{{\boldsymbol{\Gamma }}}_{t}^{(2)}f(\,-\,d),\,{\alpha }_{1}{{\bf{T}}}_{3}={{\boldsymbol{\Lambda }}}_{t}^{(2)}{Y}_{2}+{{\boldsymbol{\Gamma }}}_{t}^{(2)}{Y}_{3}$$

We can obtain ${{\bf{Q}}}_{0t},\,{{\bf{Q}}}_{1t}^{+},\,{{\bf{Q}}}_{1t}^{-}\,{\rm{and}}\,{{\bf{Q}}}_{2t}$ from (39–42) as

49 $${{\bf{Q}}}_{0t}=\frac{1}{M}\{{{\boldsymbol{{\rm T}}}}_{1}{\alpha }_{1}[({\alpha }_{1}+{\alpha }_{2})+({\alpha }_{1}-{\alpha }_{2}){e}^{-2{\alpha }_{1}d}]-2({{\boldsymbol{{\rm T}}}}_{2}{\alpha }_{2}+{{\boldsymbol{{\rm T}}}}_{3}{\alpha }_{1}){\alpha }_{1}{e}^{-{\alpha }_{1}d}\}$$

50 $${{\bf{Q}}}_{1t}^{+}=\frac{1}{M}\{{{\boldsymbol{{\rm T}}}}_{1}{\alpha }_{1}({\alpha }_{1}-{\alpha }_{2}){e}^{-2{\alpha }_{1}d}-({{\boldsymbol{{\rm T}}}}_{2}{\alpha }_{2}+{{\boldsymbol{{\rm T}}}}_{3}{\alpha }_{1})({\alpha }_{0}+{\alpha }_{1}){e}^{-{\alpha }_{1}d}\}$$

51 $${{\bf{Q}}}_{1t}^{-}=\frac{1}{M}\{{{\boldsymbol{{\rm T}}}}_{1}{\alpha }_{1}({\alpha }_{1}+{\alpha }_{2})+({{\boldsymbol{{\rm T}}}}_{2}{\alpha }_{2}+{{\boldsymbol{{\rm T}}}}_{3}{\alpha }_{1})({\alpha }_{0}-{\alpha }_{1}){e}^{-{\alpha }_{1}d}\}$$

52 $${{\bf{Q}}}_{2t}=\frac{1}{M}\{2{{\boldsymbol{{\rm T}}}}_{1}{\alpha }_{1}^{2}{e}^{-{\alpha }_{1}d}+({{\boldsymbol{{\rm T}}}}_{2}-{{\boldsymbol{{\rm T}}}}_{3}){\alpha }_{1}({\alpha }_{0}+{\alpha }_{1})+({{\boldsymbol{{\rm T}}}}_{2}+{{\boldsymbol{{\rm T}}}}_{3}){\alpha }_{1}({\alpha }_{0}-{\alpha }_{1}){e}^{-2{\alpha }_{1}d}\}{e}^{{\alpha }_{2}d}$$

with

53 $$M=({\alpha }_{0}+{\alpha }_{1})({\alpha }_{1}+{\alpha }_{2})+({\alpha }_{0}-{\alpha }_{1})({\alpha }_{1}-{\alpha }_{2}){e}^{-2{\alpha }_{1}d}$$

In the similar way, make use of the found ${{\bf{Q}}}_{0t},\,{{\bf{Q}}}_{1t}^{+},\,{{\bf{Q}}}_{1t}^{-},\,{\rm{and}}\,{{\bf{Q}}}_{2t}$ and let

54 $${\alpha }_{1}{X}_{1}=jk\hat{\eta }\cdot (\,-\,{\varepsilon }_{1r}^{\ast }{{\bf{Q}}}_{0t}+{{\bf{Q}}}_{1t}^{+}+{{\bf{Q}}}_{1t}^{-})+{{\rm{\Lambda }}}_{z}^{(1)}{Y}_{1}+{{\rm{\Gamma }}}_{z}^{(1)}{Y}_{2}$$

55 $${X}_{2}={{\rm{\Lambda }}}_{z}^{(2)}f(d)+{{\rm{\Gamma }}}_{z}^{(2)}f(\,-\,d)$$

56 $$\begin{array}{rcl}{\alpha }_{1}{X}_{3} & = & {\varepsilon }_{2r}^{\ast }\{jk\hat{\eta }\cdot ({{\bf{Q}}}_{1t}^{+}{e}^{{\alpha }_{1}d}+{{\bf{Q}}}_{1t}^{-}{e}^{-{\alpha }_{1}d}-\frac{{\varepsilon }_{1r}^{\ast }}{{\varepsilon }_{2r}^{\ast }}{{\bf{Q}}}_{2t}{e}^{-{\alpha }_{2}d})\\  &  & +jk\hat{\eta }\cdot [{{\boldsymbol{\Lambda }}}_{t}^{(2)}f(d)+{{\boldsymbol{\Gamma }}}_{t}^{(2)}f(-d)]+{{\rm{\Lambda }}}_{z}^{(2)}{Y}_{3}+{{\rm{\Gamma }}}_{z}^{(2)}{Y}_{4}\}\end{array}$$

We can obtain ${Q}_{0z},\,{Q}_{1z}^{+},\,{Q}_{1z}^{+}\,{\rm{and}}\,{Q}_{2z}$ from (43–46) as

57 $${Q}_{0z}=\frac{1}{N}\{{\alpha }_{1}{X}_{1}[({\alpha }_{1}{\varepsilon }_{2r}^{\ast }+{\alpha }_{2}{\varepsilon }_{1r}^{\ast })+({\alpha }_{1}{\varepsilon }_{2r}^{\ast }-{\alpha }_{2}{\varepsilon }_{1r}^{\ast }){e}^{-2{\alpha }_{1}d}]-2({\alpha }_{2}{\varepsilon }_{1r}^{\ast }{X}_{2}+{\alpha }_{1}{X}_{3}){\alpha }_{1}{e}^{-{\alpha }_{1}d}\}$$

58 $${Q}_{1z}^{+}=\frac{1}{N}\{{\alpha }_{1}{X}_{1}({\alpha }_{1}{\varepsilon }_{2r}^{\ast }-{\alpha }_{2}{\varepsilon }_{1r}^{\ast }){e}^{-{\alpha }_{1}d}-\,({\alpha }_{0}{\varepsilon }_{1r}^{\ast }+{\alpha }_{1})({\alpha }_{2}{\varepsilon }_{1r}^{\ast }{X}_{2}+{\alpha }_{1}{X}_{3})\}{e}^{-{\alpha }_{1}d}$$

59 $${Q}_{1z}^{-}=\frac{1}{N}\{{\alpha }_{1}{X}_{1}({\alpha }_{1}{\varepsilon }_{2r}^{\ast }+{\alpha }_{2}{\varepsilon }_{1r}^{\ast })+({\alpha }_{0}{\varepsilon }_{1r}^{\ast }-{\alpha }_{1})({\alpha }_{2}{\varepsilon }_{1r}^{\ast }{X}_{2}+{\alpha }_{1}{X}_{3}){e}^{-{\alpha }_{1}d}\}$$

60 $${Q}_{2z}=\frac{1}{N}\{2{X}_{1}{\alpha }_{1}^{2}{\varepsilon }_{2r}^{\ast }{e}^{-{\alpha }_{1}d}+{\alpha }_{1}({\varepsilon }_{2r}^{\ast }{X}_{2}-{X}_{3})({\alpha }_{0}{\varepsilon }_{1r}^{\ast }+{\alpha }_{1})+{\alpha }_{1}({\varepsilon }_{2r}^{\ast }{X}_{2}+{X}_{3})({\alpha }_{0}{\varepsilon }_{1r}^{\ast }-{\alpha }_{1}){e}^{-2{\alpha }_{1}d}\}{e}^{{\alpha }_{2}d}$$

with

61 $$N=({\alpha }_{1}+{\alpha }_{0}{\varepsilon }_{1r}^{\ast })({\alpha }_{2}{\varepsilon }_{1r}^{\ast }+{\alpha }_{1}{\varepsilon }_{2r}^{\ast })+({\alpha }_{1}-{\alpha }_{0}{\varepsilon }_{1r}^{\ast })({\alpha }_{2}{\varepsilon }_{1r}^{\ast }-{\alpha }_{1}{\varepsilon }_{2r}^{\ast }){e}^{-2{\alpha }_{1}d}$$
